# Case of Colloid Nodular Goitre in a Rare Submandibular Location

**DOI:** 10.7759/cureus.18035

**Published:** 2021-09-16

**Authors:** Varsha Rangankar, Sameeh Uz Zaman, Krishnarjun Muralinath, Viraj P Shah, Pratiksha Yadav

**Affiliations:** 1 Radiology, Dr. D.Y. Patil Medical College, Hospital and Research Center, Pune, IND

**Keywords:** ectopic thyroid, submandibular, goitre, hypothyroidism, congenital

## Abstract

Ectopic thyroid gland occurs due to aberrant descent of primitive thyroid gland to the final pre-tracheal position and failure of fusion of thyroid anlages. Submandibular ectopic thyroid is a rare thyroid anomaly that can present with or without an orthotopic thyroid gland. We present a case of a young female with hypothyroidism and left submandibular swelling demonstrated to be an ectopic thyroid with the colloid nodular goitre on imaging and cytology.

## Introduction

Thyroid gland development begins in-utero at the 20-24th day of gestation with thickening of the ventral wall endoderm of the primitive foregut which forms the medial thyroid anlage appearing as midline thyroid diverticulum in the base of the tongue at foramen cecum [1]. The lateral anlage develops from neural crest multipotent cells in the fourth pharyngeal pouch derived from ultimobranchial bodies [1,2]. During the early fifth week of gestation, median thyroid anlage traverses caudally from the foramen caecum in the pre-hyoid region along the course of the thyroglossal duct. Medial anlage which contributes to the development of the major portion of the thyroid gland fuses with the lateral thyroid anlages. The cranial part of the thyroglossal duct attenuates and disappears, while the caudal end bifurcates and forms two lobes of the thyroid [1]. The thyroid gland development and migration to its normal site anterior and lateral to the 2nd, 3rd, and 4th tracheal cartilages are completed by the seventh week of gestation. Any arrest of migration or over descent or non-fusion of the anlages causes ectopic thyroid which constitutes 48-61% of thyroid dysgenesis cases [2]. The ectopic thyroid is typically seen along the migration path of the primitive thyroid; in the lingual region, suprahyoid, hyoid, or infrahyoid suprahyoid sublingual region, and pre-laryngeal region, most common being the ectopic lingual thyroid [1-3]. Ectopic thyroid in the lateral location like the submandibular region is rarely seen [2].

The genetic research studies in the developmental anomalies of the thyroid have proposed the possible role of TITF-1 (Nkx2-1), Foxe1(TITF-2), and PAX-8 mutations [2, 4]. Significantly raised levels of TITF1/Nkx2-1 proteins are reported in ectopic thyroid as compared to normally located thyroid by some authors with a possible contribution in the abnormal function seen in ectopic thyroid [5]. The incidence of thyroid ectopia is 1/100,000 to 1/300,000 in the general population and 1/4000 to 1/8000 in population, who already have a pre-existing thyroid disorder and a 4:1 female to male preponderance [6]. Patients with thyroid ectopia may complain of various symptoms depending upon the location like local swelling, dyspnoea, and dysphagia and usually have associated hypothyroidism [5-7].

We present a rare case of ectopic thyroid in the submandibular location on the left side with colloid nodular goitre in a 19-year old female and its findings on imaging with confirmation of the diagnosis on fine-needle aspiration cytology (FNAC).

## Case presentation

**Figure 1:**
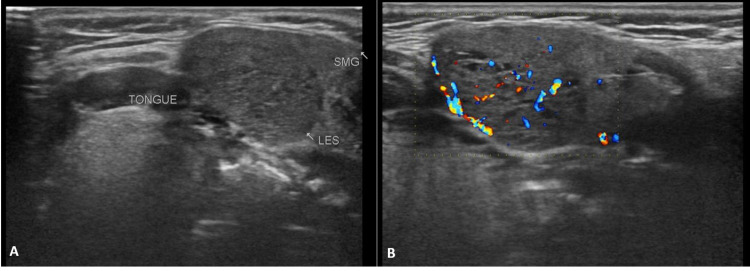
Ultrasound (USG) of the left submandibular region shows a well-defined, round to oval, heterogeneous isoechoic lesion compressing the submandibular gland with few cystic foci within (A) and increased vascularity on color Doppler (B).

A 19-year-old female reported to radiology for evaluation of palpable mass in the submandibular region on the left side. The patient had constitutional symptoms of hypothyroidism like tiredness, weight gain, constipation. She was diagnosed to have hypothyroidism based on the thyroid function test report which showed a raised thyroid-stimulating hormone (TSH) value (7.8 mIU/L) and normal triiodothyronine (T3) and thyroxine (T4 values (120.78 ng/dL and 5.97 µg/dL respectively). Ultrasound (USG) of the neck performed after she was clinically diagnosed with hypothyroidism revealed a non-visualization of the thyroid gland in the usual pre-tracheal location. A well-defined, round to oval, heterogeneous isoechoic lesion of size 25 x 18 mm was seen in the left submandibular region. The lesion had few cystic foci within and had increased vascularity on color Doppler (Figure 1).

**Figure 2:**
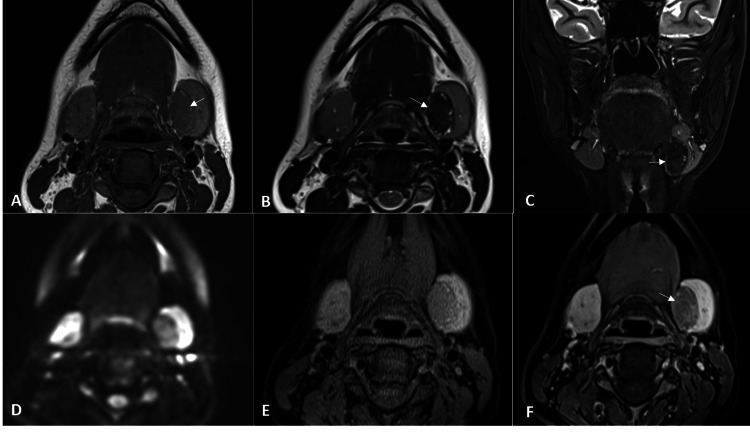
Magnetic resonance imaging (MRI) of the neck showing a well-defined, oval lesion (white arrows) in left submandibular space, appearing isointense to slightly hyperintense to muscle on T1-weighted (A) and T2-weighted images (B) and STIR images (C) with no diffusion restriction (D). The lesion showed mild to moderate enhancement on post gadolinium T1 FS images (E, F).

It was separate from, but compressing the adjacent submandibular gland. Magnetic Resonance Imaging (MRI) of the neck was performed for the characterisation of the lesion. MRI (Figure 2) disclosed a well-demarcated oval lesion of size 25 x 23 x 13 mm in the left submandibular region. The lesion appeared isointense to slightly hyperintense to muscle on T1-weighted and T2-weighted images and showed isointense to slightly hyperintense signals on Short Tau Inversion Recovery (STIR) images with no diffusion restriction. The lesion showed mild to moderate enhancement on post gadolinium fat-saturated T1-weighted images. Multiple small cystic foci were seen in the lesion. The lesion was seen to be causing compression and lateral displacement of the left submandibular gland. Medially, the lesion was seen abutting the mylohyoid, hyoglossus muscle, and digastric muscle with maintained fat planes.

**Figure 3:**
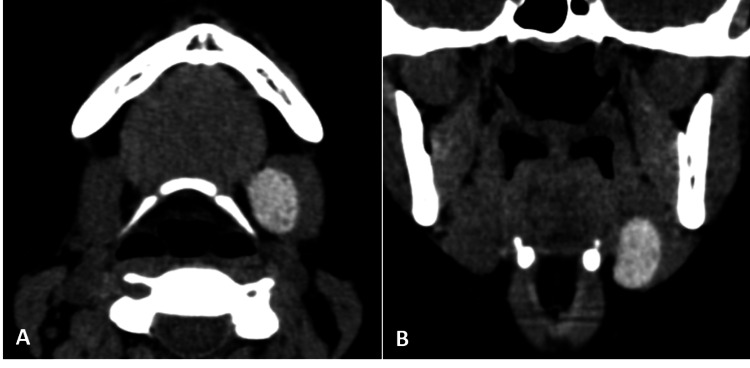
(A, B): CT images showing a well-defined, oval, homogenously hyperdense lesion in the left submandibular region compressing the submandibular gland and displacing it laterally.

**Figure 4:**
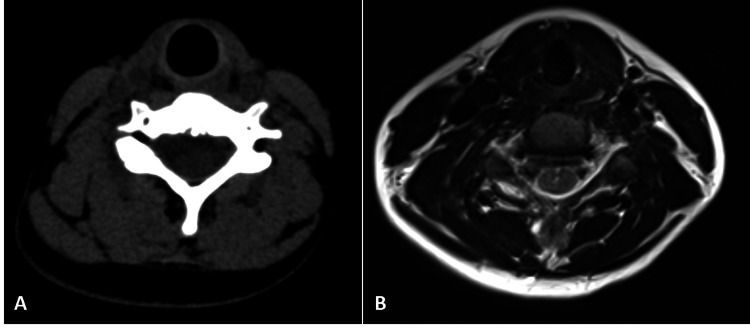
Non-visualisation of the thyroid gland in its normal anatomical position on CT (A) and MRI (B) images.

Non-contrast enhanced Computed Tomography (NCCT) of the neck region was performed to collaborate the MRI findings and screen for ectopic thyroid tissue in any other location in the neck. NCCT (Figure 3) demonstrated an oval, well-defined, homogenously hyperdense lesion of size 25 x 23 x14 mm, in the left submandibular region compressing and displacing the submandibular gland laterally. The thyroid gland was not seen in its normal pre-tracheal place on CT and MRI studies (Figure 4).

The submandibular lesion was thought to represent an ectopic thyroid gland with cystic degeneration given its morphology and non-visualisation normal thyroid gland. In our case to confirm the diagnosis, USG-guided FNAC was performed and aspirate was taken from the left submandibular lesion in view of the thyroid function test and imaging findings. The cytology of the aspirate revealed a hemorrhagic background with scanty colloid material, within which were abundant clusters of thyroid follicular epithelial cells arranged in cohesive monolayered sheets, along with many bare nuclei and few foamy macrophages. These features were suggestive of ectopic submandibular thyroid with colloid nodular goitre. As the patient was diagnosed with hypothyroidism, levothyroxine 50 µg (Thyronorm by Abbott, Mumbai, India) once daily was prescribed.

The limitation in our case was that a technetium or iodine thyroid nuclear scan was not performed for confirmation of diagnosis and to detect ectopic thyroid tissue at any other location. Though the screening NCCT did not reveal hyperdense thyroid tissue at normal or ectopic location except the left submandibular region.

## Discussion

The thyroid gland, situated anterior and lateral to the second to fourth tracheal cartilages, is composed of the two lobes connected by the isthmus. Thyroid ectopia is defined as the existence of thyroid tissue in a location that is different from its normal anatomical site. Ectopic thyroid reported in 48 to 61% of all cases is the most frequently encountered type of thyroid developmental anomaly [8]. The normal thyroid gland may or may not co-exist with the ectopic thyroid and is rarely present in cases of the lateral ectopic thyroid gland [3,8]. Thyroid ectopia usually occurs along the descent of primitive thyroid at the endodermal origin at foramen cecum to the infrahyoid region along the thyroglossal duct. It can also occur in regions apart from the path of the normal thyroid gland descent, like the palatine tonsils, parotid gland, iris, axilla, breast, intrathoracic and intra-abdominal organs [5-10]. Lingual thyroid is the most common and reported in up to 70 to 90 percent of all cases of ectopic thyroid [3,9].

Submandibular ectopic thyroid is very rare and is thought to occur when lateral thyroid anlage fails to migrate and fuse with the median anlage at the seventh week of gestation [11]. However, some authors have found abnormal descent of the median anlage to be a more likely reason for submandibular ectopic thyroid [2]. It has been reported in the literature to be more common in females and shows right laterality [6,11-12].

Clinical features can range from asymptomatic mass in the neck to stomatolalia, dysphagia, dysphonia, and dyspnoea based on the location, extent, and size of the ectopic thyroid [6-13]. Differentials for ectopic thyroid gland depend on the location and in our case of submandibular location, they are - minor salivary gland tumors, hyperplastic lymphoid tissue, lymphoma, sebaceous cysts, adenoma, fibroma, lipoma, lymphangioma, squamous cell carcinoma, and vascular tumors [3,6,12].

The benign and malignant conditions occurring in the ectopic thyroid gland are similar to those seen in a normal thyroid gland. The cases of lateral ectopic thyroid with orthoptic thyroid can present with pathological changes in either or both the thyroid tissues [12]. The ectopic thyroid gland can present with hyperthyroidism or hypothyroidism but is more commonly associated with hypothyroidism which is seen in 33-62% cases of ectopic thyroid [6,13-15].

The malignant conditions in ectopic thyroid occur very rarely accounting for about 1% of cases with papillary thyroid carcinoma being the most common [11]. The only definite way to rule out malignancy and diagnose ectopic thyroid is by histopathology. However, modalities like USG, CT, and MRI are helpful to know the size, shape, appearance, location, and extent of the lesion. Scintigraphy using iodine-123 or Technetium-99m pertechnetate offer high sensitivity and specificity in detecting functioning thyroid cells in the ectopic thyroid gland and also to detect the presence of any functioning thyroid gland at the normal location.

Management of patients with ectopic thyroid depends on the age and symptoms of the patient, thyroid function, and associated comorbidities. Surgical excision of the ectopic gland is recommended in patients with hyperthyroidism. Debulking with thyroxine therapy may be needed in euthyroid and hypothyroid patients having local symptoms in the form of mass effect and airway compression. The patient in our case was diagnosed with hypothyroidism due to colloid nodular goitre in the submandibular ectopic thyroid. No surgical interventional was done at the time of diagnosis and the patient has been prescribed levothyroxine for hypothyroidism.

## Conclusions

Ectopic thyroid gland in the submandibular region is a rare developmental thyroid anomaly that can be associated with or without an orthotopic thyroid gland. It can present as a palpable mass and show pathologies similar to the orthotopic thyroid gland including hyperplasia, goitre, and rarely malignancy with significant clinical implications. Hypothyroidism is more frequently present in cases of ectopic thyroid requiring levothyroxine supplement. A multimodality imaging approach and FNAC is often required for the diagnosis of the submandibular ectopic thyroid gland.
